# Ultra-Deep Bisulfite Sequencing to Detect Specific DNA Methylation Patterns of Minor Cell Types in Heterogeneous Cell Populations: An Example of the Pituitary Tissue

**DOI:** 10.1371/journal.pone.0146498

**Published:** 2016-01-11

**Authors:** Yoshikazu Arai, Hisho Fukukawa, Takanori Atozi, Shoma Matsumoto, Yutaka Hanazono, Hiroshi Nagashima, Jun Ohgane

**Affiliations:** 1 Laboratory of Genomic function Engineering, Department of Life Sciences, School of Agriculture, Meiji University, Kawasaki, Japan; 2 Division of Regenerative Medicine, Center for Molecular Medicine, Jichi Medical University, Tochigi, Japan; 3 CREST, Japan Science and Technology Agency, Tokyo, Japan; 4 Laboratory of Developmental Engineering, Department of Life Sciences, School of Agriculture, Meiji University, Kawasaki, Japan; 5 Meiji University International Institute for Bio-Resource Research (MUIIBR), Kawasaki, Japan; University of Navarra, SPAIN

## Abstract

DNA methylation is an epigenetic modification important for cell fate determination and cell type-specific gene expression. Transcriptional regulatory regions of the mammalian genome contain a large number of tissue/cell type-dependent differentially methylated regions (T-DMRs) with DNA methylation patterns crucial for transcription of the corresponding genes. In general, tissues consist of multiple cell types in various proportions, making it difficult to detect T-DMRs of minor cell types in tissues. The present study attempts to detect T-DMRs of minor cell types in tissues by ultra-deep bisulfite sequencing of cell type-restricted genes and to assume proportions of minor cell types based on DNA methylation patterns of sequenced reads. For this purpose, we focused on transcriptionally active hypomethylated alleles (Hypo-alleles), which can be recognized by the high ratio of unmethylated CpGs in each sequenced read (allele). The pituitary gland contains multiple cell types including five hormone-expressing cell types and stem/progenitor cells, each of which is a minor cell type in the pituitary tissue. By ultra-deep sequencing of more than 100 reads for detection of Hypo-alleles in pituitary cell type-specific genes, we identified T-DMRs specific to hormone-expressing cells and stem/progenitor cells and used them to estimate the proportions of each cell type based on the Hypo-allele ratio in pituitary tissue. Therefore, introduction of the novel Hypo-allele concept enabled us to detect T-DMRs of minor cell types with estimation of their proportions in the tissue by ultra-deep bisulfite sequencing.

## Introduction

Epigenetic systems, mainly consisting of DNA methylation and histone modifications, are crucial for cell type-specific gene expression [1–5]. Transcriptional regulatory regions of the mammalian genome contain a large number of tissue-dependent differentially methylated regions (T-DMRs) with DNA methylation patterns that determine expression states of the corresponding genes [6,7]. The DNA methylation status of T-DMRs changes dynamically during embryonic development by *de novo* methylation and demethylation related to cell fate determination [6,8]. Thus, the DNA methylation status of T-DMRs is a useful index for the identification of cell types.

Many methods for analyzing DNA methylation have been developed, and bisulfite sequencing is particularly useful for analyzing DNA methylation status in transcriptional regulatory regions since it can determine DNA methylation status at single-CpG resolution within bisulfite PCR products [9,10]. Thus, this method effectively identifies T-DMRs of gene loci by comparing DNA methylation status across tissue/cell types [11]. The DNA methylation rate is calculated based on the number of methylated CpGs in 10–20 clones of bisulfite PCR products analyzed by Sanger sequencing. For DNA methylation analysis of heterogeneous cell populations such as tissues consisting of multiple cell types, this method for calculating DNA methylation rates most likely reflects the major cell populations. In contrast, the DNA methylation status of minor cell types is difficult to detect with conventional bisulfite sequencing analysis of tissues.

Our previous study of porcine induced pluripotent stem cells (iPSCs) indicated that pluripotency-related genes such as *Sall4* exhibit both hypomethylated and hypermethylated sequenced clones by Sanger sequencing of 10–20 clones [12]. This is likely because porcine iPSCs consist of mixed cell populations of properly and improperly reprogrammed cells [13]. Consistent with this, properly reprogrammed iPSCs exhibited more hypomethylated sequenced clones than less-reprogrammed cells [12]. In bisulfite DNA methylation analysis by Sanger sequencing, each sequenced clone effectively represents one allele in the analyzed cell population or tissue. This suggests that the methylation status of each clone reflects a hypo- or hypermethylated allele in one cell, enabling us to identify cell type-specific DNA methylation patterns in heterogeneous cell populations or tissues by focusing on the CpG methylation pattern of each sequenced clone. Except for unconventional genes exhibiting biased allele usages in each cell such as imprinted genes and female X-linked genes, the proportion of a hypomethylated allele (Hypo-allele) for a cell type-specific gene in a tissue reflects that of the corresponding cell type in the tissue. However, typical T-DMR searches from multiple tissues by Sanger sequencing are performed using only 10–20 clones of bisulfite PCR products per tissue that are equivalent to 10–20 alleles/cells in the tissue, making it difficult to identify specific DNA methylation patterns of minor cell types. Therefore, analysis of DNA methylation patterns of minor cell types in tissues requires many more clones of bisulfite PCR products, but the cost and labor of sequencing a large number of clones for multiple tissues is prohibitive using conventional Sanger sequencing.

Recent technological progress has developed various next-generation sequencing techniques that can be used to sequence tens of millions of DNA fragments at a time, and these technologies are applicable to epigenetic research such as whole-genome bisulfite DNA methylation analysis [14–17]. However, the DNA methylation level of each CpG is determined by several tens of reads or less in these whole-genome bisulfite sequencing analyses, equivalent to analyzing several tens of clones by conventional bisulfite DNA methylation analysis of PCR products with Sanger sequencing, and is not sufficient and accurate enough to detect the DNA methylation status of minor cell types in a tissue. Given these considerations, we applied ultra-deep bisulfite sequencing of cell type-restricted genes to detect DNA methylation patterns of minor cell types in tissues and calculate the proportions of minor cell types focusing on the DNA methylation pattern of each sequenced read, especially on transcriptionally active Hypo-alleles that can be recognized by the high ratio of unmethylated CpGs in each sequenced read (allele).

Pituitary is a key endocrine tissue with various vital physiological functions including growth, reproduction, and metabolism. The pituitary gland is composed of three anatomical parts: the anterior lobe, the intermediate lobe, and the neural lobe. The anterior lobe consists of heterogeneous cell populations comprising five hormone-expressing cell types: growth hormone (Gh)-producing somatotroph, prolactin (Prl)-producing mammotroph, thyroid-stimulating hormone (Tsh)-producing thyrotroph, luteinizing hormone (Lh)- and follicle-stimulating hormone (FSH)-producing gonadotroph, and adrenocorticotropic hormone (Acth)-producing corticotroph [18–20]. Pituitary tissue also contains stem/progenitor cells that can differentiate into hormone-producing cells during pituitary morphogenesis and pituitary cell regeneration. Each hormone-producing cell displays cell type-specific gene expression patterns. Therefore, pituitary tissue is a good model for validation of our approach for DNA methylation-based cell type identification.

Herein, we analyzed the DNA methylation patterns of cell type-restricted genes in pig pituitary tissue containing multiple cell types including various hormone-expressing cells as well as stem/progenitor cells.

## Materials and Methods

### Genomic DNA extraction and PCR amplification

Genomic DNA extraction and bisulfite conversion were carried out as described previously [12]. Pituitary glands used in this study were purchased from Funakoshi Co., Ltd. (Tokyo, Japan). Genomic DNA was purified from porcine pituitary gland, brain, liver, and porcine fetal fibroblast (PFF) by Proteinase K digestion and PCI (phenol, chloroform, and isoamyl alcohol: 50/49/1, v/v) extraction followed by ethanol precipitation. Genomic DNA was also purified from the porcine iPSC line (Porco Rosso-4) [12] as a proof-of-principle. Whole pituitary tissue from a single pig was ground into a fine and well-mixed powder with a mechanical homogenizer (Automill TK-AM5-S, Tokken, Inc., Chiba, Japan) to avoid affecting cell type proportions before proceeding to genomic DNA purification. Purified genomic DNA was restriction-digested with *Hind*III (TaKaRa, Kyoto, Japan) and purified by ethanol precipitation. After denaturing digested genomic DNA with 0.3 M NaOH, sodium metabisulfite (pH 5.0) and hydroquinone were added to final concentrations of 2.0 M and 0.5 mM, respectively. The bisulfite reaction was performed using a PCR machine as follows: 20 cycles of 95°C for 30 s and 55°C for 15 min, followed by 55°C for 10 h. Bisulfite-treated genomic DNA was purified with a QIAquick Gel Extraction Kit (Qiagen GmbH, Hilden, Germany), desulfonated with 0.3 M NaOH at 37°C for 15 min, and ethanol precipitated. Purified bisulfite-treated DNA was amplified with BioTaq HS DNA polymerase (BIOLINE, London, UK) using specific primers for T-DMRs (S1 Table). In the PCR products of the 40 genes (average product size: 412 bp), the average number of CpG and the average distance between two neighboring CpGs were 10 CpGs (ranging between 3 and 26) and 27 bp (ranging between 1 and 243), respectively. PCR reactions were performed under the following conditions: 95°C for 10 min; 40 cycles of 95°C for 30 s, 60°C for 30 s, and 72°C for 1 min; final extension 72°C for 2 min. PCR products were used to prepare the sample library for Illumina MiSeq sequencing or conventional subcloning into a pGEM-T Easy vector (Promega, Madison, WI) for sodium bisulfite sequencing using a capillary sequencer.

### Library preparation for next-generation sequencing

Libraries were prepared using the TruSeq PCR-Free Sample Prep kit (Illumina, Inc., San Diego, CA, USA). Briefly, 2 μg pooled PCR amplicons purified by gel filtration was treated with T4 polynucleotide kinase (TaKaRa) at 37°C for 60 min and purified using Agencourt AMPure XP magnetic beads (Beckman Coulter, Pasadena, CA, USA). A-tailing and adapter ligation were performed using the manufacturer’s protocol, and adapter-ligated DNA was further purified using Agencourt AMPure XP magnetic beads. Concentrations of the purified DNA fragments were measured using Qubit dsDNA BR assay kit on a Qubit 2.0 Fluorometer (Invitrogen, Rockville, MD, USA). Prepared libraries were sequenced with an Illumina MiSeq using the MiSeq Reagent Kit v3 (600 cycles) (Illumina). The sequencing data were deposited in DDBJ with accession number DRA004190. After sequencing, FASTAQ-formatted sequence data were mapped on the genomic sequences of PCR amplicons using Bismark [21].

### RNA extraction and gene expression by RT-PCR

Total RNA was extracted using TRIzol reagent (Invitrogen). After treatment with RNase-free DNase I (Invitrogen), first-strand cDNA synthesis was performed using random hexamers and a Superscript III First-strand Synthesis System (Invitrogen). PCR was performed using BioTaq HS DNA polymerase with specific primers for gene loci (S1 Table). PCR reactions were performed under the following conditions: 95°C for 10 min; 30 cycles of 95°C for 30 s, 60°C for 30 s, and 72°C for 1 min; final extension 72°C for 2 min.

## Results

### Validation of Hypo-allele detection by ultra-deep bisulfite sequencing using next-generation sequencing

**Fig 1:**
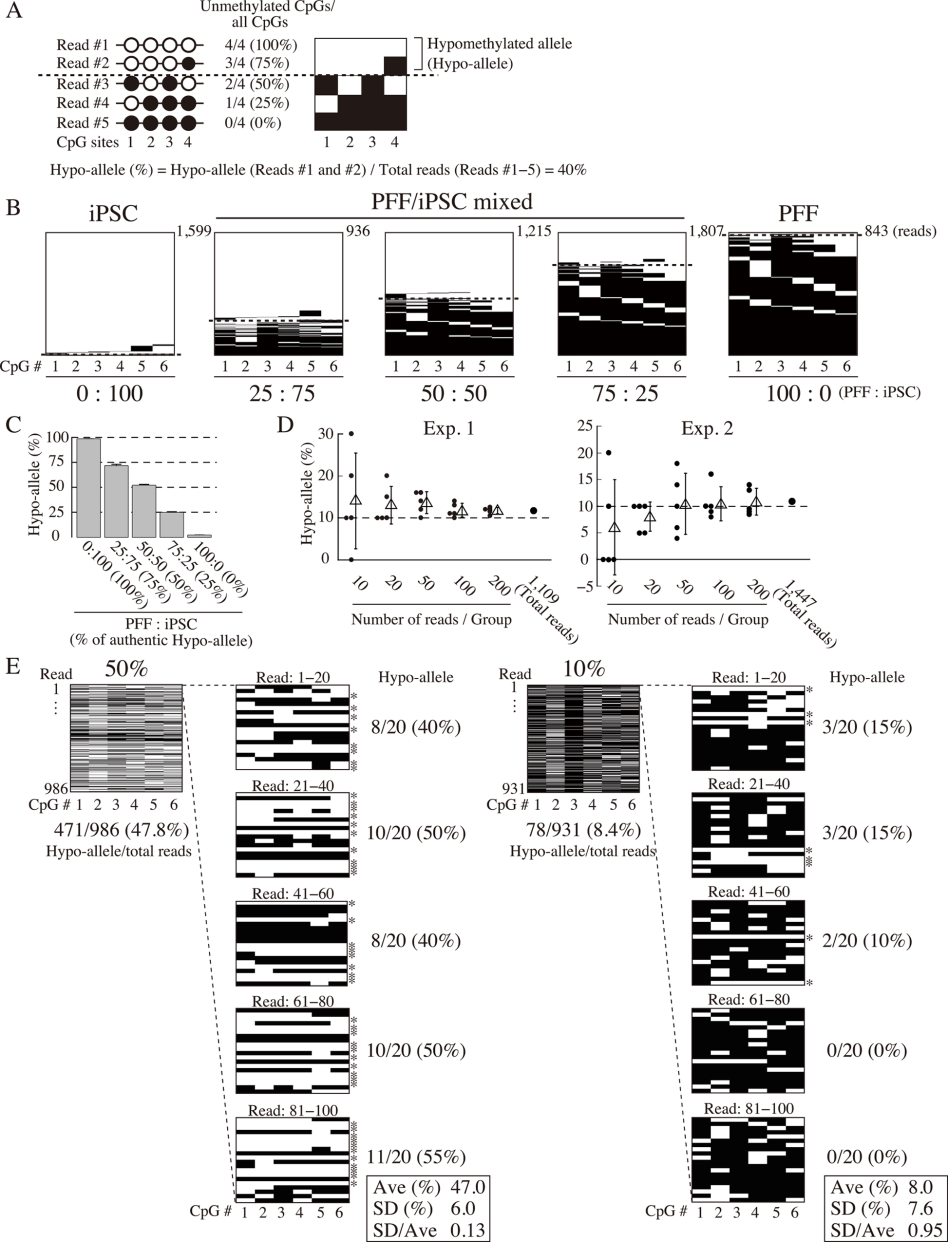
Validation of hypomethylated alleles (Hypo-alleles) by MiSeq ultra-deep sequencing. ≥75% unmethylated CpG sites (3 or 4 out of the 4 CpGs shown) are defined as Hypo-alleles. Among the five reads, reads 1 and 2 are Hypo-alleles, and the Hypo-allele ratio is 40% (2 reads/5 reads). Left panel: conventional CpG methylation analysis data; white and black circles indicate unmethylated and methylated CpGs, respectively. Right panel: MiSeq ultra-deep bisulfite analysis; white and black bars indicate unmethylated and methylated CpGs, respectively. (B) Validation of Hypo-allele ratio analysis by MiSeq ultra-deep bisulfite sequencing at the *Sall4* T-DMR using mixtures of genomic DNAs from PFF and iPSC. Sequenced reads above the dotted lines are Hypo-alleles. White and black bars indicate unmethylated and methylated CpGs, respectively. Mixtures of genomic DNAs of PFF and iPSC (0:100, 25:75, 50:50, 75:25, or 100:0) would exhibit the expected respective Hypo-allele ratios (100%, 75%, 50%, 25%, or 0%) for the *Sall4* T-DMR. (C) Hypo-allele ratios of the *Sall4* T-DMR analyzed by ultra-deep sequencing. Hypo-allele ratios were calculated from the PFF/iPSC mixtures (expected Hypo-allele ratios of 100%, 75%, 50%, 25%, and 0%) shown in Fig 1B. The Hypo-allele ratios from three independent experiments are shown as mean ± SE (n = 3). (D) Accuracy of detecting Hypo-allele ratio (10%) in relation to sequenced read numbers based on the MiSeq data at the *Sall4* T-DMR. For this analysis, genomic DNAs of PFF and iPSC were mixed at 90:10 for a Hypo-allele ratio of 10%. From two independent MiSeq analyses, 1,109 reads (Exp. 1) and 1,447 reads (Exp. 2) were obtained. Hypo-allele ratios of each trial are plotted (filled circles, n = 5), and mean ± SD for five trials each for 10, 20, 50, 100, or 200 reads are plotted as triangles with lines. (E) Examination of detection accuracy of Hypo-allele (10% or 50%) by conventional analysis of small numbers of sequencing reads based on the MiSeq data at the *Sall4* T-DMR. Genomic DNAs of PFF and iPSC were mixed at 90:10 or 50:50 for samples exhibiting Hypo-allele ratios of 10% or 50%, respectively. From the raw data, 20 successive reads were grouped from the first through 100th read. The Hypo-allele ratios of 20-read groups were calculated. Asterisks indicate Hypo-alleles. White and black bars indicate unmethylated CpGs and methylated CpGs, respectively.

In the present study, we attempted to detect the DNA methylation status of cell type-specific genes, interpreting each sequenced read of DNA methylation data with our novel Hypo-allele approach instead of the conventional methylation rate method. We defined sequenced reads with ≥75% unmethylated CpGs as Hypo-alleles (Fig 1A). In the first experiment using a next-generation sequencer, we tested detection of Hypo-alleles from minor cell populations experimentally created by mixing genomic DNA from two cell types at various ratios before bisulfite treatment.

In our previous study on porcine iPSCs, *Sall4*, a pluripotency-related gene, was identified with a T-DMR that was almost fully methylated in PFFs but almost fully unmethylated in properly reprogrammed iPSCs [12]. We mixed genomic DNA from PFFs and iPSCs at different ratios (0:100, 25:75, 50:50, 75:25, and 100:0) expected to exhibit Hypo-allele rates of 100, 75, 50, 25, and 0%, respectively, at the *Sall4* T-DMR. Determination of *Sall4* Hypo-alleles was based on the methylation status of six CpG sites identified as the T-DMR within the seven CpGs of the bisulfite PCR product [12]. We defined sequenced reads with 5–6 unmethylated CpGs in the PCR product as Hypo-alleles (≥75% of CpGs unmethylated). MiSeq sequencing revealed decreasing Hypo-allele ratios at the *Sall4* T-DMR with increasing PFF genomic DNA (Fig 1B and 1C), and the Hypo-allele percentages were close to the expected values (R^2^ = 0.998). In contrast, conventional analysis (methylated CpGs/all CpGs) resulted in estimation of lower DNA methylation levels than expected, especially for samples with higher levels of PFF genomic DNA (S1 Fig). Although the *Sall4* locus was hypermethylated in PFF, a small proportion of CpGs was detected as unmethylated [12]. Therefore, the Hypo-allele ratio indicates the DNA methylation status of the *Sall4* T-DMR more accurately than the conventional DNA methylation rate.

In addition, we defined two alternate criteria for Hypo-alleles, all (100%) or ≥50% of CpGs unmethylated, and calculated Hypo-allele ratios from the data used in Fig 1B (S2 Fig). When Hypo-alleles were defined as 100% unmethylated CpGs, the Hypo-allele ratio was underestimated, especially for the samples with higher levels of iPSC genomic DNA, compared with the authentic Hypo-allele ratio. In contrast, when Hypo-alleles were defined as ≥50% unmethylated CpGs, the Hypo-allele ratios were overestimated for samples with higher levels of PFF genomic DNA. Therefore, in the present study, we defined sequenced reads with ≥75% unmethylated CpGs as Hypo-alleles.

We next examined the relationship between sequenced read number and Hypo-allele ratio. A genomic DNA sample with a 10% Hypo-allele ratio at the *Sall4* T-DMR was experimentally prepared by mixing PFF and iPSC genomic DNAs (90:10) and analyzed by MiSeq ultra-deep bisulfite sequencing. Using the raw data for the sample, designated numbers of successive reads (10, 20, 50, 100, or 200) were grouped as individual trials for Hypo-allele ratio calculation, and the trials were performed five times (Fig 1D). For example, in the 10-read trial, reads 1–10, 11–20, 21–30, 31–40, and 41–50 were grouped as trials for Hypo-allele ratio calculations. From two independent experiments on the sample with 10% Hypo-allele (Exps. 1 and 2), the Hypo-allele ratios (%) calculated exhibited considerable variations depending on the grouped read number (10, 20, 50, 100, or 200), especially for fewer reads (10, 20, and 50). Variations in Hypo-allele ratio (%) were smaller for groups of 100 or 200 reads, and their average ratios were close to the authentic Hypo-allele ratio of 10%. The average Hypo-allele ratios (%) with standard deviations of the five calculations were 11.8 ± 1.6% (Exp. 1) and 10.4 ± 3.2% (Exp. 2) for the 100-read groups, and 11.7 ± 0.8% (Exp. 1) and 10.8 ± 2.5% (Exp. 2) for the 200-read groups. The total numbers of *Sall4* T-DMR reads were 1,109 and 1,447 in Exps. 1 and 2, respectively, and the Hypo-allele ratios (%) calculated from the total number of reads were 11.7% (Exp. 1) and 10.8% (Exp. 2), clearly indicating that the average Hypo-allele ratios (%) of the 100- and 200-read groups were close to those of the total sequenced reads and the authentic Hypo-allele ratio. These data strongly suggest that at least 100 reads (clones) are required to reproducibly detect a Hypo-allele ratio of 10% (equivalent to 10% active alleles/cells in a tissue) by conventional bisulfite sequencing analysis.

Another experimentally prepared sample with a 50% Hypo-allele ratio (50:50 PFF and iPSC genomic DNA) yielded an average Hypo-allele ratio of 47.0 ± 6.0%, and the ratio of standard deviation to mean (SD/Ave) was 0.13, within the range recognized as accurate, by conventional bisulfite sequencing of 20 reads (clones) with five replicates (Fig 1E, left panel). This indicates that moderate levels of Hypo-allele ratios around 50% can be reliably detected by conventional bisulfite sequencing analysis of a few tens of reads (clones). On the other hand, the average Hypo-allele ratio was 8.0 ± 7.6% for the analysis of the experimentally prepared 10% Hypo-allele sample (Fig 1E, right panel), and SD/Ave was 0.95, which could mean the standard deviation was too large compared with the average Hypo-allele ratio. Thus, consistent with the results of Fig 1D, the samples with lower Hypo-allele ratios yielded less accurate Hypo-allele ratio estimates by conventional bisulfite sequencing analysis of a few tens of reads (clones). Collectively, DNA methylation analysis using next-generation sequencing was more accurate for more than 100 reads, especially for T-DMRs with lower Hypo-allele ratios. Therefore, we decided to analyze T-DMRs with more than 100 reads obtained by MiSeq ultra-deep bisulfite sequencing.

### Validation of Hypo-allele detection for endogenous genes

**Fig 2:**
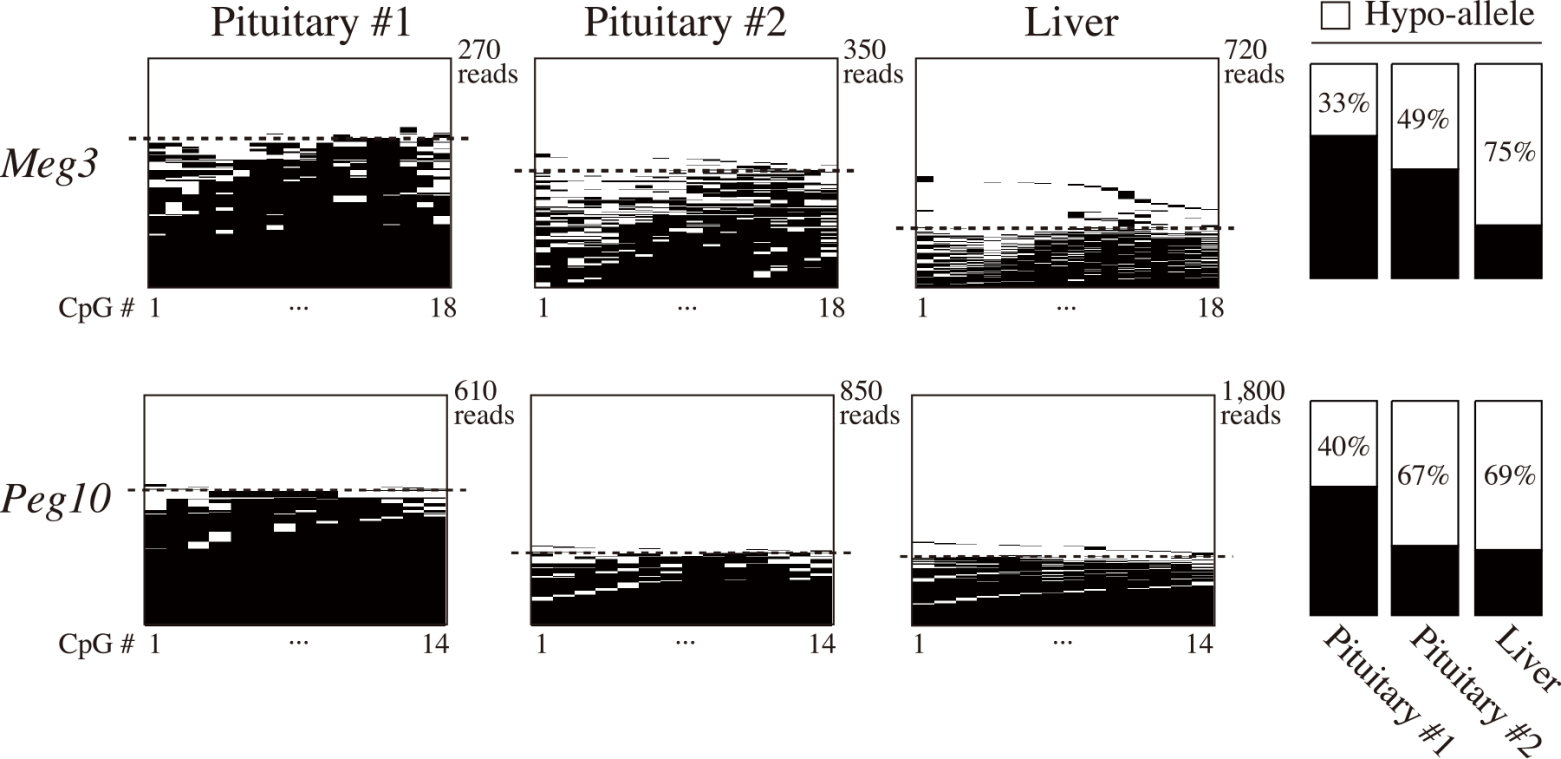
Detection of Hypo-alleles of endogenous imprinted genes in porcine pituitary and liver. Bisulfite ultra-deep sequencing was performed for differentially methylated regions of imprinted *Meg3* and *Peg10* genes, and their Hypo-allele ratios were calculated as in Fig 1. Sequenced reads above the dotted lines are Hypo-alleles. White and black bars indicate unmethylated and methylated CpGs, respectively.

To test the reproducibility of Hypo-allele detection of endogenous genes in physiological conditions, we next analyzed differentially methylated regions (DMRs) of imprinted porcine genes *Meg3* and *Peg10* by MiSeq ultra-deep sequencing. Previous reports found different methylation and expression patterns of these two genes between the paternal and maternal alleles [22,23]. In this study, Hypo-alleles were clearly observed by next-generation sequencing with ratios around 50% in two porcine pituitary samples (#1 and #2) and liver (Fig 2). Although the paternal and maternal alleles of these two genes could not be identified due to lack of informative single-nucleotide polymorphisms in the sequenced regions, these results indicate that hypomethylated and active alleles of imprinted genes in physiological samples are also detectable at around 50% by our ultra-deep bisulfite sequencing analysis.

### DNA methylation analysis of pituitary-related genes using next-generation sequencing

**Fig 3:**
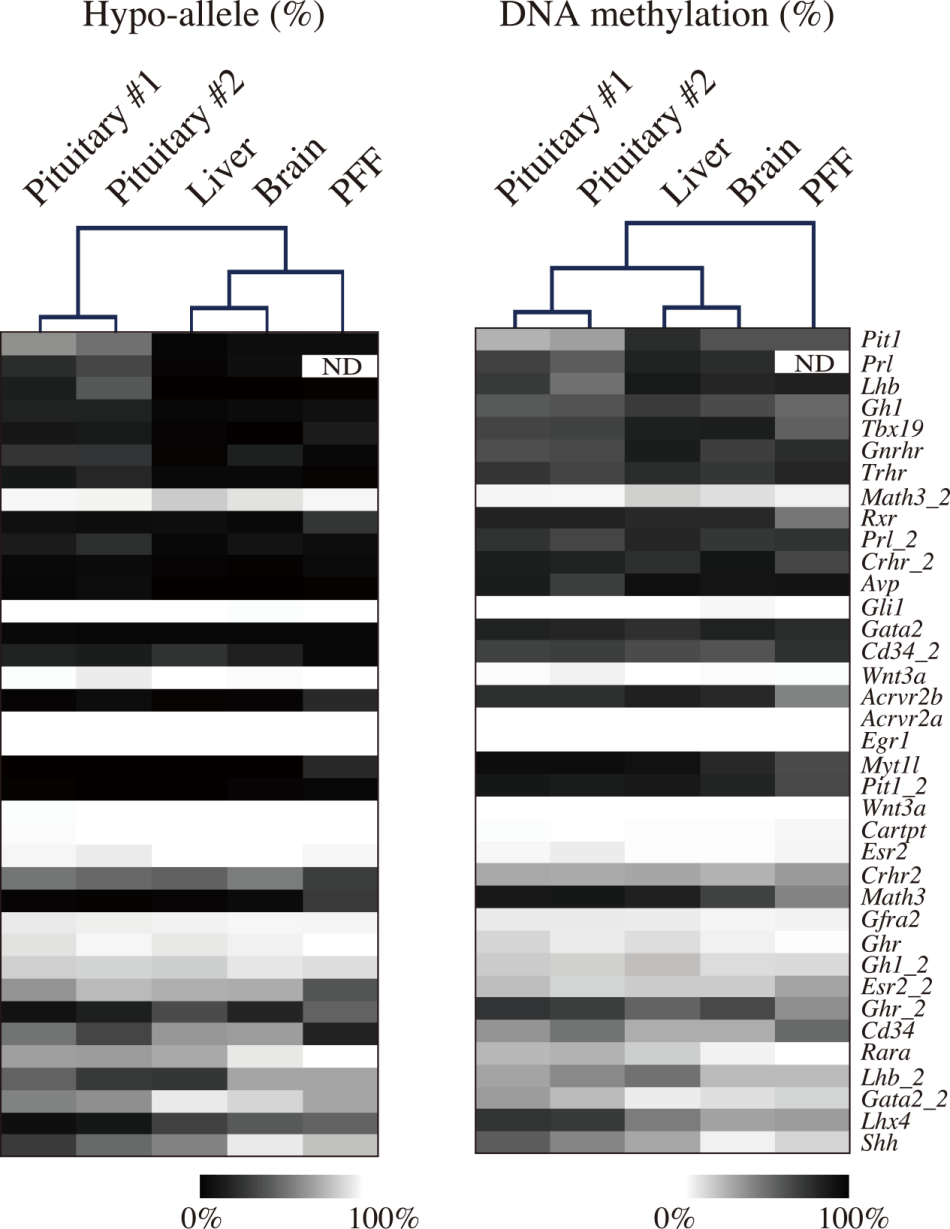
Comparison of DNA methylation profiles of pituitary-related genes in porcine tissues by ultra-deep bisulfite sequencing between the conventional DNA methylation analysis and a novel Hypo-allele ratio analysis. Hypo-allele ratios of 37 pituitary-related genes were analyzed in pituitary (#1 and #2), liver, brain, and PFF samples using a MiSeq sequencer. The Hypo-allele data for each tissue are shown as a heatmap after hierarchical clustering based on Euclidean distance (left panel). Using the same bisulfite sequencing data, the conventional DNA methylation degrees calculated by methyl-CpGs/total CpGs are also shown as a heatmap with hierarchical clustering (right panel). ND, No data.

To determine T-DMRs of genes related to pituitary function and development, we selected 37 annotated porcine gene loci and analyzed the DNA methylation status of transcriptional regulatory regions using a next-generation sequencer (Fig 3). Using five tissues/cells (pituitary #1 and #2, liver, brain, and PFF), the average number of sequenced reads from the 37 genes was 3,832−6,321 (S2 Table), and 25 out of 37 genes gave more than 1,000 reads in all five tissues/cells. The Hypo-allele ratios (%) obtained by next-generation sequencing are shown as a heatmap (Fig 3), and hierarchical clustering analysis was performed. The two pituitary samples (#1 and #2) reproducibly exhibited similar Hypo-allele ratio patterns, and the samples were clearly divided into different branches (Fig 3, left panel). Pituitary tissue shared a branch with liver and brain samples when compared with PFF by conventional DNA methylation analysis based on DNA methylation level (%), although the pituitary-related genes were selected and analyzed (Fig 3, right panel). These results suggest that employing the Hypo-allele ratio for characterizing pituitary tissue using pituitary-related genes gives more accurate results than conventional DNA methylation analysis based on DNA methylation level.

**Fig 4:**
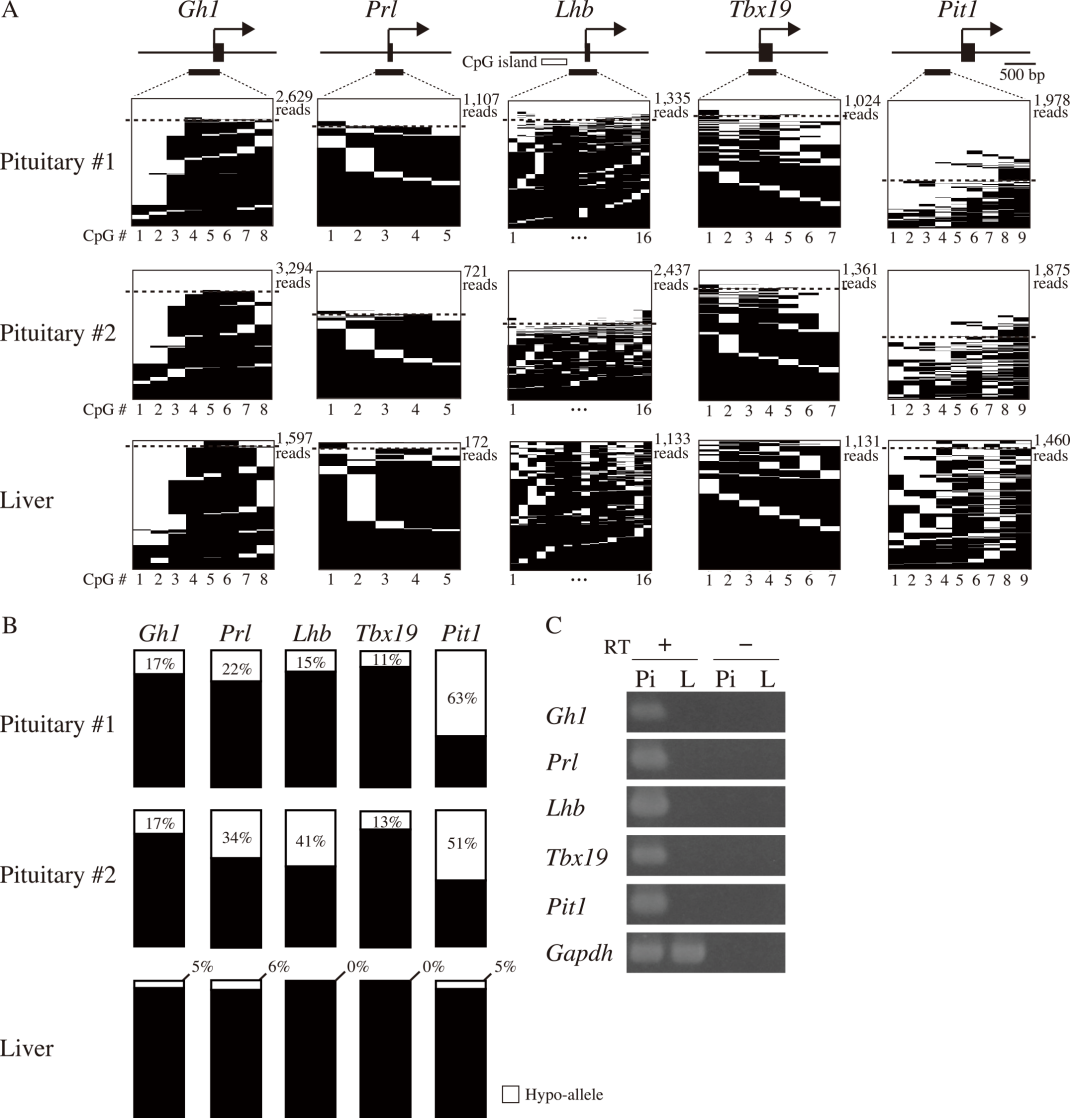
Hypo-allele analysis of five T-DMRs of pituitary cell type-restricted genes. (A) *Gh1*, *Prl*, *Lhb*, *Tbx19*, and *Pit1* were analyzed using MiSeq sequencing. Horizontal lines indicate each sequenced read with methylation statuses of unmethylated (white) or methylated (black) CpGs. Sequenced reads above the dotted lines indicate Hypo-alleles. The numbers below the panels indicate CpG positions in bisulfite PCR products. (B) Summary of Hypo-allele (%) based on the MiSeq data of Fig 4A. (C) Expression levels of mRNAs of the five pituitary cell type-restricted genes and an internal control *Gapdh* gene in pituitary (Pi) and liver (Li).

We next investigated the Hypo-allele ratios of T-DMRs within transcriptional regulatory regions of five pituitary-related genes (Fig 4A). *Tbx19* and *Pit1* are expressed in the progenitor of hormone-producing cells: Tbx19-positive cells differentiate to corticotrophs, and Pit1-positive cells differentiate to somatotrophs, lactotrophs, or gonadotrophs [24,25]. DNA methylation analysis of the five genes by next-generation sequencing revealed that Hypo-alleles were barely detected in liver (0–6%), whereas higher levels (11−63%) of Hypo-alleles were observed in both of the pituitary samples (#1 and #2) (Fig 4B and S3 Table). In addition, these pituitary-related genes were expressed in the pituitary gland but not in the liver (Fig 4C), suggesting that Hypo-alleles detected in pituitary tissue correlated with their gene expression. In contrast, there was no clear difference in DNA methylation levels for the T-DMRs of the five pituitary-related genes between pituitary and liver samples using the conventional calculation (methylated CpGs/all CpGs) except for *Pit1* (S3 Fig). Hypo-allele percentages of *Prl* and *Lhb* were different between pituitary #1 and #2. Pituitary #1 and #2 were derived from female and male pigs, respectively, and the male was castrated immediately after birth. Lack of sex hormone secretion from the male gonads may change the populations of Prl- and Lhb-producing cells in pituitary tissue.

### Detection of Hypo-alleles by Sanger sequencing

**Fig 5:**
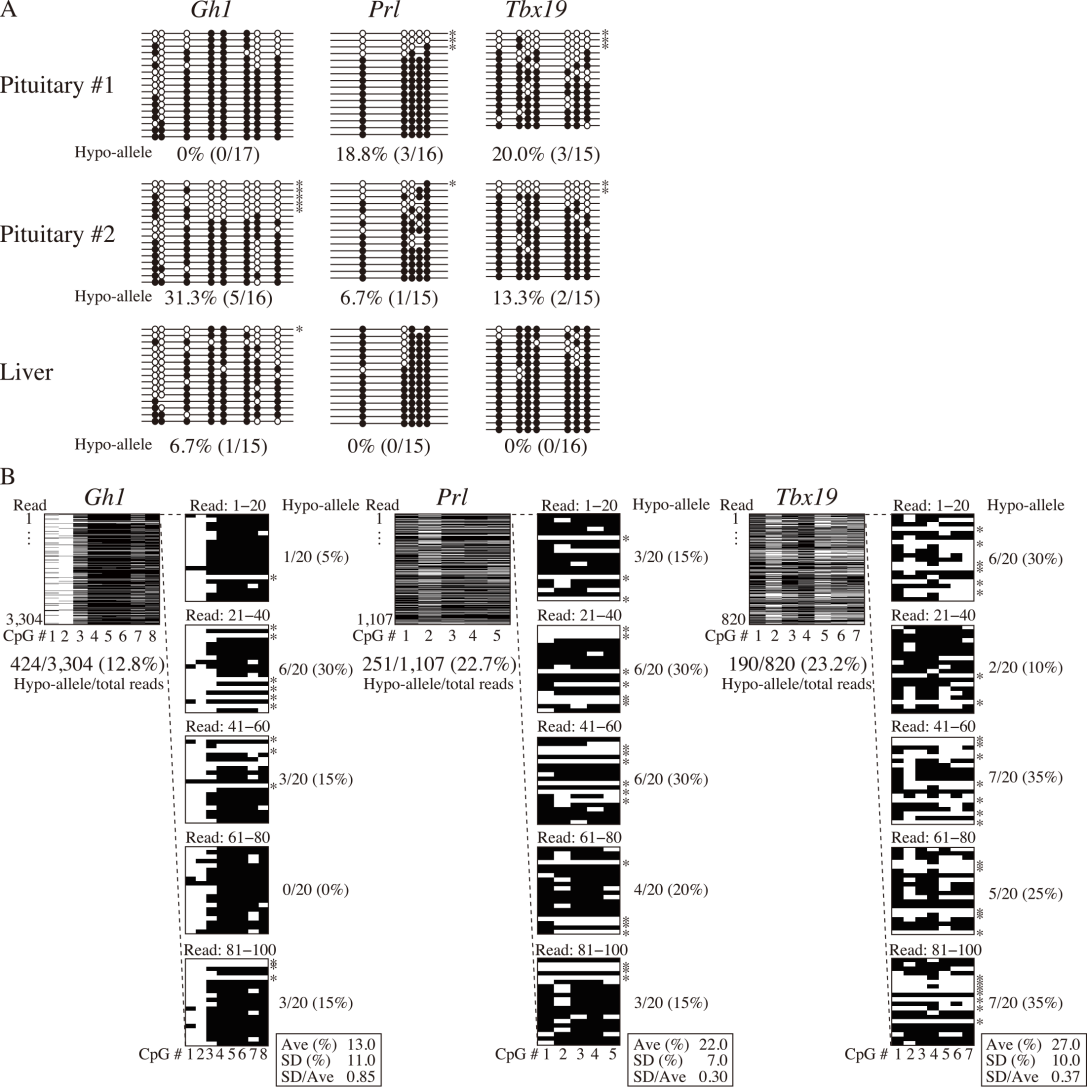
Bisulfite analyses of relatively small numbers of clones (reads) for detection of Hypo-alleles by Sanger and MiSeq sequencing. Cell type-restricted genes (*Gh1*, *Prl*, and *Tbx19*) were analyzed by Sanger sequencing of 15–20 clones. Black and white circles indicate methylated and unmethylated CpGs, respectively. (B) Successive 20 reads were grouped as a trial and analyzed, and five trials were performed from the first read of MiSeq raw data of the *Gh1*, *Prl*, and *Tbx19* T-DMRs to mimic the conventional bisulfite sequencing analyses of 20 clones (reads) five times each. The Hypo-allele ratio of the *Gh1* T-DMR from the 3,304 total reads was 12.8%, whereas those of five groups of 20 reads were between 0–30% (average: 13.0%, SD: 11.0%). Similarly, the Hypo-allele ratios of the *Prl* and *Tbx19* T-DMRs from the 1,107 and 820 total reads were 22.7% and 23.2%, respectively, whereas those of five trials of 20 reads were between 15–30% (average: 22.0%, SD: 7.0%) for the *Prl* T-DMR and 10–35% (average: 27.0%, SD: 10.0%) for the *Tbx19* T-DMR. Methylated and unmethylated CpGs are shown as black and white bars, respectively. Asterisks indicate Hypo-alleles.

In this study, we used Hypo-allele data from over 100 reads using next-generation sequencing. We compared our results with those of conventional Sanger sequencing using about 20 clones for each sample and analyzed Hypo-allele ratios of the three pituitary-related genes (*Gh1*, *Prl*, and *Tbx19*) (Fig 5A). At the *Gh1* locus, the Hypo-allele ratio was 17% for pituitary #1 by next-generation sequencing, whereas Hypo-alleles were not detected by Sanger sequencing of 17 clones. In addition, the percentages of Hypo-alleles at the *Prl* locus of pituitaries #1 and #2 by Sanger sequencing (18.8% and 6.7%, respectively) was lower than those by ultra-deep sequencing (22% and 34%, respectively; Fig 4), suggesting that Hypo-allele data from Sanger sequencing of about 20 clones exhibited lower resolution and higher variance than ultra-deep sequencing data of about 1,000 reads with MiSeq as estimated in Fig 1D and 1E. Furthermore, DNA methylation levels (methylated CpGs/all CpGs) obtained by Sanger sequencing did not different between pituitary and liver samples (S4 Fig).

To examine whether Hypo-allele detection depends on differences between the capillary sequencer and next-generation sequencer, we next analyzed Hypo-alleles with successive groups of 20 reads from MiSeq raw data as with PFF/iPSC mixture analysis in Fig 1E. The *Gh1* T-DMR of pituitary #1 exhibited varied Hypo-allele ratios among the five trials, and the average Hypo-allele ratio (%) and SD/Ave of the five trials of the 20 reads were 13 ± 11% and 0.85, respectively (Fig 5B, left panel). Hypo-allele percentages were also varied among the five trials of the *Prl* and *Tbx19* T-DMRs, and the average Hypo-allele ratio (%) and SD/Ave were 22 ± 7% and 0.30 for the *Prl* T-DMR (middle panel) and 27 ± 10% and 0.37 for the *Tbx19* T-DMR (right panel) (Fig 5B). These results suggest that detection of Hypo-alleles with 15−20 sequenced reads for pituitary-related genes expressed from minor pituitary cell types is not sufficiently accurate even using next-generation sequencing. Thus, ultra-deep bisulfite sequencing that enables analysis of hundreds of reads for each locus is a useful approach for determining T-DMRs in the genes expressed in minor cell types.

## Discussion

Previous studies on T-DMR identification specific to certain tissues or cultured cells were performed based on methylation ratios calculated from the number of methylated CpGs out of the total number of CpGs in a few tens of sequenced PCR products. Since a tissue generally consists of multiple cell types with various proportions, conventional T-DMR searches are effective for genes that are active in a major cell type in the tissue but cannot easily detect genes that are active in minor cell types. This is likely because each cell has two alleles of each gene, and alleles of minor cell types have stochastically lower chances of being sequenced than major cell types when only a few tens of clones are selected for bisulfite sequencing analysis. In addition, a previous study demonstrated the difficulty in detecting relatively subtle differences in methylation levels for small numbers of clones [26]. To our knowledge, the present study is the first to focus on hypomethylation of each sequenced read (transcriptionally active allele) obtained by ultra-deep bisulfite sequencing of cell type-restricted genes, which can reliably detect hypomethylation of minor cell types by sequencing hundreds or thousands of bisulfite PCR fragments of cell type-restricted genes. By applying this concept for analysis of cell type-specific DNA methylation in the pituitary tissue, we identified hypomethylated T-DMRs of hormone-expressing cells as Hypo-alleles. Thus, our method can effectively detect T-DMRs of minor cell types by focusing on DNA methylation profiles of Hypo-alleles.

In general, 10–20 clones (reads) of PCR-amplified DNA fragments are analyzed in conventional bisulfite sequencing. In the present study, bisulfite-sequenced loci with moderate ratios of Hypo-alleles reproducibly exhibited actual Hypo-allele ratios from five trials successively grouping 20 reads between the first and 100th read of MiSeq raw data, which are equivalent to five trials of conventional bisulfite analyses of 20 clones by Sanger sequencing. However, bisulfite-sequenced loci with relatively low ratios of Hypo-alleles did not exhibit authentic Hypo-allele ratios from a few tens of reads (clones) by either MiSeq or Sanger sequencing. In contrast, ultra-deep sequencing of hundreds to thousands of reads was required to reliably detect low ratios of Hypo-alleles for cell type-restricted genes. This is consistent with our pilot experiment indicating that at least 100–200 reads are required to accurately detect experimental pools with 10% *Sall4* T-DMR Hypo-alleles. These results indicate that ultra-deep sequencing with a large number of reads is required to detect Hypo-alleles of minor cell types in tissues.

Pituitary-specific hormone genes are active only in the corresponding hormone-expressing cell types such as somatotrophs and lactotrophs, each of which is a minor cell type in pituitary tissue [24,25]. Thus, conventional calculation of DNA methylation rates by methylated CpGs/total CpGs for promoter regions of pituitary-specific hormone genes did not exhibit obvious differences, resulting in failure of T-DMR identification between pituitary and other tissues by both MiSeq ultra-deep sequencing and Sanger sequencing. Therefore, identification of T-DMRs for cell type-specific genes active only in minor cell populations must combine ultra-deep next-generation sequencing and the Hypo-allele approach focusing on DNA methylation patterns of each sequenced read (allele).

**Fig 6:**
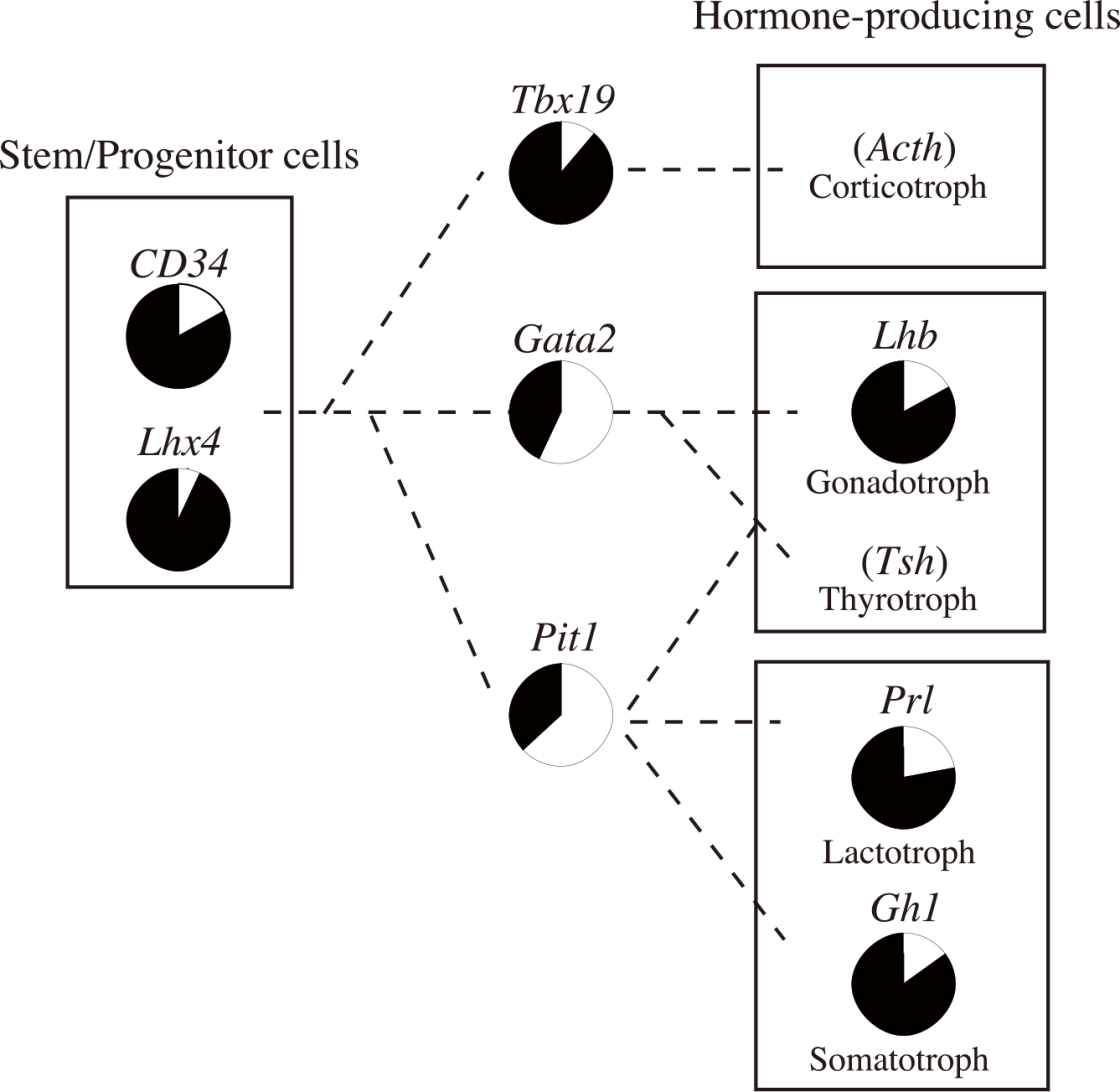
Schematic diagram of predicted proportions of pituitary cell types in adult porcine pituitary tissue. Proportions of several pituitary cell types were estimated based on the Hypo-allele ratios of the pituitary cell type-restricted genes (white).

Each read of a cell type-specific gene by MiSeq ultra-deep sequencing reflects the methylation pattern of one allele (e.g., hypomethylated allele) in a cell amplified by bisulfite PCR, leading us to hypothesize that the Hypo-allele ratio of a non-imprinted and autosomal gene can be used to estimate the proportion of the cell type in the tissue. In addition, narrow but deep sequencing by MiSeq is advantageous for detecting specific DNA methylation patterns of minor cell types in tissues as discussed above, whereas wide but shallow analysis such as whole-genome bisulfite sequencing would be more suitable for detection of DNA methylation patterns of major cell types in tissues. As summarized in Fig 6, the proportions of stem/progenitor and hormone-expressing cell types could be calculated based on these Hypo-allele ratios of marker genes of the cell types. Previous work showed that the ratios of Gh1-, Prl-, and Lhb-positive cells in rat pituitary tissue are 40%, 30%, and 10%, respectively [18]. Consistent with this finding, our data suggest that 20–40% of cells are prone to express *Gh1*, *Prl*, or *Lhb* in porcine pituitary tissue. In addition, the stem/progenitor cell populations were ~10% based on the Hypo-allele ratios of *Lhx4* or *CD34* T-DMRs. Although the proportions of each cell type in porcine pituitary tissue are currently unknown, a single porcine pituitary gland provides sufficient genomic DNA for simultaneous MiSeq ultra-deep bisulfite analysis of hundreds of T-DMRs in contrast to those of mice and rats, which require pooling to obtain a sufficient amount of genomic DNA. In addition, this concept of Hypo-allele can be further applied to other tissues, cancers, or heterogeneous cell populations to identify minor cell types in these samples. However, estimation of minor cell types based on the Hypo-allele ratio requires information on cell type-specific gene expression patterns. Therefore, genes with confirmed cell type-specific expression patterns need to be analyzed. To identify proportions of minor cell types in other sample types using our Hypo-allele concept, analysis of DNA methylation and gene expression (cell type specificity) by *in situ* hybridization or immunohistochemistry using neighboring regions of the samples used for DNA methylation analysis should be effective. Therefore, our present study indicated that DNA methylation profiles focusing on DNA methylation patterns of each sequencing read (allele) are useful for estimating proportions of various porcine pituitary cell types.

In conclusion, introduction of the novel Hypo-allele concept herein enabled us to detect T-DMRs of minor cell types and their proportions in porcine pituitary tissue by bench-top MiSeq next-generation sequencing.

## Supporting Information

S1 Fig Conventional calculation of DNA methylation levels by methyl-CpGs/total CpGs. MiSeq sequencing data for the mixtures of PFF and iPSC shown in Fig 1 were analyzed based on conventional DNA methylation levels calculated by methyl-CpGs/total CpGs. The methylation degrees are shown as mean ± SE (n = 3).
(PDF)

S2 Fig Hypo-allele ratios of the *Sall4* T-DMR based on two different definitions of Hypo-alleles. Sequenced reads with 100% or ≥50% unmethylated CpGs were tentatively defined as Hypo-alleles, and the Hypo-allele ratios using the two definitions were calculated from the MiSeq data for mixtures of PFF and iPSC genomic DNAs analyzed in Fig 1B. Hypo-allele ratios from three independent experiments are shown as mean ± SE (n = 3).
(PDF)

S3 Fig DNA methylation levels of pituitary cell type-restricted genes (*Gh1*, *Prl*, *Lhb*, *Tbx19*, and *Pit1*) in pituitary (#1 and #2) and liver samples. The methylation data shown in Fig 4 using a next-generation sequencer were recalculated as conventional DNA methylation degrees using the following formula: methyl-CpGs/total CpGs. Experiments were performed twice independently.
(PDF)

S4 Fig DNA methylation levels of pituitary cell type-restricted genes (*Gh1*, *Prl*, *Tbx19*) in pituitary (#1 and #2) and liver samples. The methylation data shown in Fig 5 using Sanger sequencing were recalculated as conventional DNA methylation degrees using the following formula: methyl-CpGs/total CpGs.
(PDF)

S1 Table PCR primers. (PDF)

S2 Table Summary of MiSeq sequencing data. *The 37 pituitary-related genes with 100 or more reads obtained in each of the triplicate MiSeq sequencing runs were selected for DNA methylation analysis. For PFF, 36 genes out of the 37 pituitary-related genes gave reproducible sequencing data, and the 36 genes were used for DNA methylation analysis. **Mean ± SE.
(PDF)

S3 Table Summary of Hypo-allele analysis of the five T-DMRs of pituitary cell type-restricted genes shown in Fig 4. The numbers of reads and Hypo-allele ratios of two pituitary (#1 and #2) and one liver sample from two independent experiments (Exps. 1 and 2) are described for the five genes.
(PDF)

## References

[1] Shiota K. DNA methylation profiles of CpG islands for cellular differentiation and development in mammals. Cytogenet Genome Res. 2004; 105: 325–334. 15237220 10.1159/000078205

[2] Lieb JD, Beck S, Bulyk ML, Farnham P, Hattori N, Henikoff S, et al Applying whole-genome studies of epigenetic regulation to study human disease. Cytogenet Genome Res. 2006; 114: 1–15. 16717444 10.1159/000091922 PMC2734277

[3] Golob JL, Paige SL, Muskheli V, Pabon L, Murry CE. Chromatin remodeling during mouse and human embryonic stem cell differentiation. Dev Dyn. 2008; 237: 1389–1398. 10.1002/dvdy.21545 18425849 PMC3075915

[4] Ohgane J, Yagi S, Shiota K. Epigenetics: the DNA methylation profile of tissue-dependent and differentially methylated regions in cells. Placenta. 2008; Suppl A: S29–35. 18031808 10.1016/j.placenta.2007.09.011

[5] Ikegami K, Ohgane J, Tanaka S, Yagi S, Shiota K. Interplay between DNA methylation, histone modification and chromatin remodeling in stem cells and during development. Int J Dev Biol. 2009; 53: 203–214. 10.1387/ijdb.082741ki 19412882

[6] Shiota K, Kogo Y, Ohgane J, Imamura T, Urano A, Nishino K, et al Epigenetic marks by DNA methylation specific to stem, germ and somatic cells in mice. Genes Cells. 2002; 7: 961–969. 12296826 10.1046/j.1365-2443.2002.00574.x

[7] Yagi S, Hirabayashi K, Sato S, Li W, Takahashi Y, Hirakawa T, et al DNA methylation profile of tissue-dependent and differentially methylated regions (T-DMRs) in mouse promoter regions demonstrating tissue-specific gene expression. Genome Res. 2010; 18: 1969–1978. 10.1101/gr.074070.107 PMC2593572 18971312

[8] Sakamoto H, Suzuki M, Abe T, Hosoyama T, Himeno E, Tanaka S, et al Cell type-specific methylation profiles occurring disproportionately in CpG-less regions that delineate developmental similarity. Genes Cells. 2007; 10: 1123–1132. 10.1111/j.1365-2443.2007.01120.x 17903172

[9] Frommer M, McDonald LE, Millar DS, Collis CM, Watt F, Grigg GW, et al A genomic sequencing protocol that yields a positive display of 5-methylcytosine residues in individual DNA strands. Proc Natl Acad Sci U S A. 1992; 89: 1827–1831. 1542678 10.1073/pnas.89.5.1827 PMC48546

[10] Feil R, Charlton J, Bird AP, Walter J, Reik W. Methylation analysis on individual chromosomes: improved protocol for bisulphite genomic sequencing. Nucleic Acids Res. 1994; 22: 695–696. 8127720 10.1093/nar/22.4.695 PMC307863

[11] Imamura T, Ohgane J, Ito S, Ogawa T, Hattori N, Tanaka S, et al CpG island of rat sphingosine kinase-1 gene: tissue-dependent DNA methylation status and multiple alternative first exons. Genomics. 2001; 76: 117–125. 11560121 10.1006/geno.2001.6607

[12] Arai Y, Ohgane J, Fujishiro SH, Nakano K, Matsunari H, Watanabe M, et al DNA methylation profiles provide a viable index for porcine pluripotent stem cells. Genesis. 2013; 51: 763–776. 10.1002/dvg.22423 23913699 PMC4237151

[13] Fujishiro SH, Nakano K, Mizukami Y, Azami T, Arai Y, Matsunari H, et al Generation of naive-like porcine-induced pluripotent stem cells capable of contributing to embryonic and fetal development. Stem Cells Dev. 2013; 22: 473–482. 10.1089/scd.2012.0173 22889279 PMC3549629

[14] Bock C, Tomazou EM, Brinkman AB, Müller F, Simmer F, Gu H, et al Quantitative comparison of genome-wide DNA methylation mapping technologies. Nat Biotechnol. 2010; 28: 1106–1114. 10.1038/nbt.1681 20852634 PMC3066564

[15] Harris RA, Wang T, Coarfa C, Nagarajan RP, Hong C, Downey SL, et al Comparison of sequencing-based methods to profile DNA methylation and identification of monoallelic epigenetic modifications. Nat Biotechnol. 2010; 28: 1097–1105. 10.1038/nbt.1682 20852635 PMC2955169

[16] Serre D, Lee BH, Ting AH. MBD-isolated Genome Sequencing provides a high-throughput and comprehensive survey of DNA methylation in the human genome. Nucleic Acids Res. 2010; 38: 391–399. 10.1093/nar/gkp992 19906696 PMC2811030

[17] Weng X, Zhou D, Liu F, Zhang H, Ye J, Zhang Z, et al DNA methylation profiling in the thalamus and hippocampus of postnatal malnourished mice, including effects related to long-term potentiation. BMC Neurosci. 2014; 15: 31 10.1186/1471-2202-15-31 24555847 PMC3941971

[18] Ben-Jonathan N, Peleg E, Hoefer MT. Optimization of culture conditions for short-term pituitary cell culture. Methods Enzymol. 1983; 103: 249–257. 6321888 10.1016/s0076-6879(83)03016-5

[19] Zhu X, Gleiberman AS, Rosenfeld MG. Molecular physiology of pituitary development: signaling and transcriptional networks. Physiol Rev. 2007; 87: 933–963. 17615393 10.1152/physrev.00006.2006

[20] Vankelecom H, Gremeaux L. Stem cells in the pituitary gland: A burgeoning field. Gen Comp Endocrinol. 2010; 166: 478–488. 10.1016/j.ygcen.2009.11.007 19917287

[21] Krueger F, Andrews SR. Bismark: a flexible aligner and methylation caller for Bisulfite-Seq applications. Bioinformatics. 2011; 27: 1571–1572. 10.1093/bioinformatics/btr167 21493656 PMC3102221

[22] Takada S, Paulsen M, Tevendale M, Tsai CE, Kelsey G, Cattanach BM, et al Epigenetic analysis of the Dlk1-Gtl2 imprinted domain on mouse chromosome 12: implications for imprinting control from comparison with Igf2-H19. Hum Mol Genet. 2002; 11: 77–86. 11773001 10.1093/hmg/11.1.77

[23] Ono R, Shiura H, Aburatani H, Kohda T, Kaneko-Ishino T, Ishino F. Identification of a large novel imprinted gene cluster on mouse proximal chromosome 6. Genome Res. 2003; 13: 1696–1705. 12840045 10.1101/gr.906803 PMC403743

[24] Li S, Crenshaw EB 3rd, Rawson EJ, Simmons DM, Swanson LW, Rosenfeld MG. Dwarf locus mutants lacking three pituitary cell types result from mutations in the POU-domain gene pit-1. Nature. 1990; 347: 528–533. 1977085 10.1038/347528a0

[25] Lamolet B, Pulichino AM, Lamonerie T, Gauthier Y, Brue T, Enjalbert A, et al A pituitary cell-restricted T box factor, Tpit, activates POMC transcription cooperation with Pitx homeoproteins. Cell. 2001; 104: 849–859. 11290323 10.1016/s0092-8674(01)00282-3

[26] Morrill BH, Cox L, Ward A, Heywood S, Prather RS, Isom SC. Targeted DNA methylation analysis by high throughput sequencing in porcine peri-attachment embryos. J Reprod Dev. 2013; 59: 314–320. 23428632 10.1262/jrd.2012-144 PMC3934139

