# Supplementary figures and images for: Ultra-Deep Bisulfite Sequencing to Detect Specific DNA Methylation Patterns of Minor Cell Types in Heterogeneous Cell Populations: An Example of the Pituitary Tissue
Source: PLoS One. 2016 Jan 11;11(1):e0146498. doi: 10.1371/journal.pone.0146498 (PMC4709138; doi:10.1371/journal.pone.0146498)

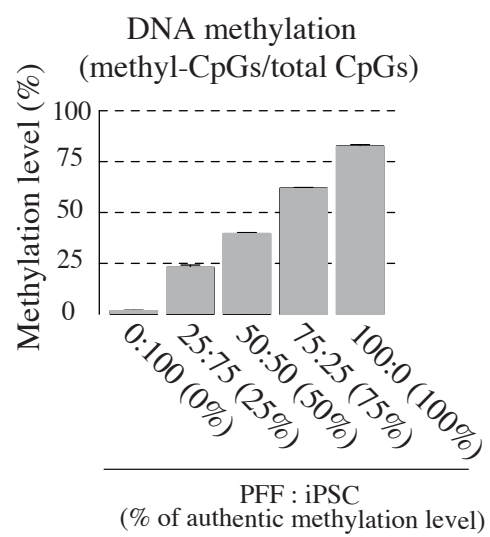

S1 Fig

Supplement: S1 Fig — MiSeq sequencing data for the mixtures of PFF and iPSC shown in Fig 1 were analyzed based on conventional DNA methylation levels calculated by methyl-CpGs/total CpGs. The methylation degrees are shown as mean ± SE (n = 3). (PDF) [file pone.0146498.s001.pdf]

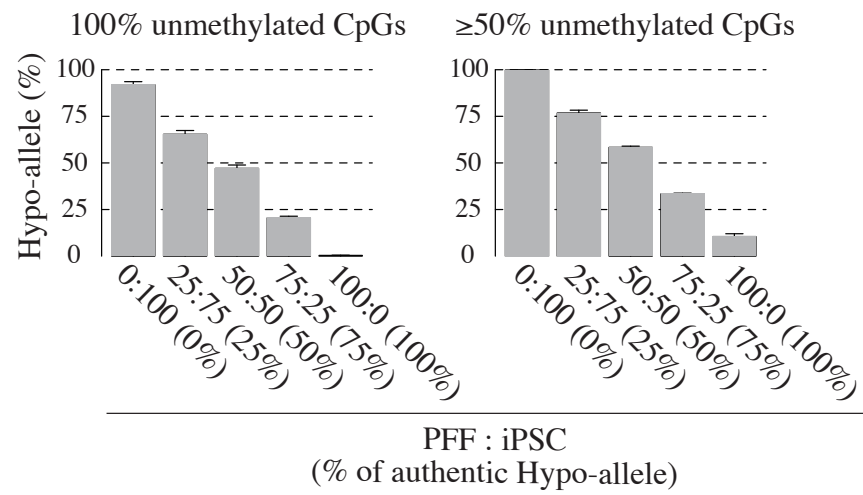

S2 Fig

Supplement: S2 Fig — Sequenced reads with 100% or ≥50% unmethylated CpGs were tentatively defined as Hypo-alleles, and the Hypo-allele ratios using the two definitions were calculated from the MiSeq data for mixtures of PFF and iPSC genomic DNAs analyzed in Fig 1B. Hypo-allele ratios from three independent experiments are shown as mean ± SE (n = 3). (PDF) [file pone.0146498.s002.pdf]

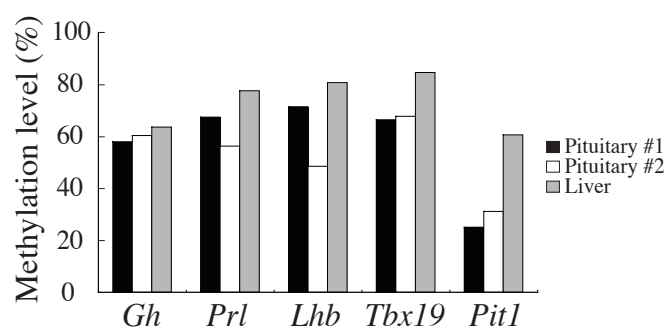

S3 Fig

Supplement: S3 Fig — The methylation data shown in Fig 4 using a next-generation sequencer were recalculated as conventional DNA methylation degrees using the following formula: methyl-CpGs/total CpGs. Experiments were performed twice independently. (PDF) [file pone.0146498.s003.pdf]

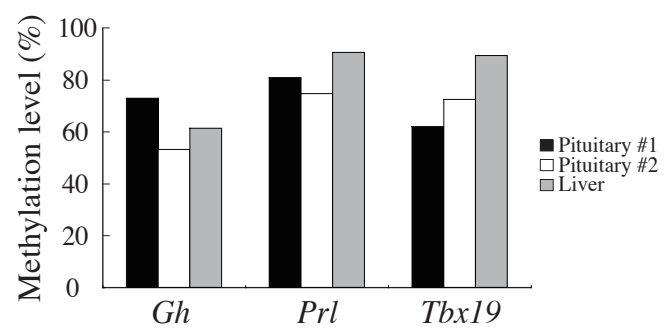

S4 Fig

Supplement: S4 Fig — The methylation data shown in Fig 5 using Sanger sequencing were recalculated as conventional DNA methylation degrees using the following formula: methyl-CpGs/total CpGs. (PDF) [file pone.0146498.s004.pdf]
