# Supplementary material for: Ultra-Deep Bisulfite Sequencing to Detect Specific DNA Methylation Patterns of Minor Cell Types in Heterogeneous Cell Populations: An Example of the Pituitary Tissue
Source: PLoS One. 2016 Jan 11;11(1):e0146498. doi: 10.1371/journal.pone.0146498 (PMC4709138; doi:10.1371/journal.pone.0146498)
Supplement: S1 Table — (PDF) [file pone.0146498.s005.pdf]

S1 Table. PCR primers.

	Bisulfite PCR primers			
	Forward prrimer (5' -3')	Reverse prrimer (5' -3')	Size (bp)	From transcription start site
Acvr2a	AATATTGTTGGATATTGAAGTTGAAG	TCTAAAAACCTCCATAAATTTTTCTAA	400	0.5 kbp downstream
Acvr2b	GGTGAGGTAGAGAGGGATTGG	TTCCTTTTATTAACATACCTACCAACA	458	1.5 kbp downstream
Avp	GATTTGGGTTTAGGGTTGGATTA	AAAATCCTCCATCAAAACTACCC	395	0.5 kbp downstream
Cartpt	TGGTTGTTGTTTTTGTTAGGTGT	CAAATACTCACCTCCAACCCTTAT	473	0.5 kbp downstream
Cd34	ATAGGGGTTTTGTAAGGGGAAA	CCCCTCTCCAACCTACAAACTA	405	1.0 kbp upstream
Cd34_2	GAGAGTTGATTTGAGGTTTAGATTTT	CCACAAAACCCCCTTCTACC	455	2.0 kbp downstream
Crhr2	TGAGGTTATGAGGTTGGGTTTT	TCCTAACTCCTCCCCCTAACA	319	3.0 kbp upstream
Crhr2_2	GTGGGTTTAGGGTGGTTAAATGT	TCATCCCTACCCCCTAACTTCTA	432	0.5 kbp upstream
Egr1	GGAGAGGGGATTTTTAGAGGGTA	ACCTACTTCTCCTCCATCTACCA	357	1.0 kbp upstream
Esr2	GTTGGGGATTGGGTGATTTA	CAAAAACCTCCCCTTCCTTTTA	468	0.5 kbp upstream
Esr2_2	GGAATAGAATTATTTTGGGAAGAGT	CTCCTCTTCTAAAAACCCACAAAA	425	0.5 kbp downstream
Gata2	TTTGATTGTGGGAGAGGTTGT	CAAAATCCCTAAACCAAAAACA	428	1.5 kbp upstream
Gata2_2	GGGGTGAGGGAGGTTATAGAGT	AAAATCCCCTCCAAACCTTATC	467	1.0 kbp downstream
Gfra2	GTATTTTTATGTTGGGTGGGGTAT	AACTCCAATCCCCAAACCTT	441	2.0 kbp upstream
Gh1	ATTAGTGTAGGGAGGAGTAAAGGTGTAA	ATCCACAAATCCCTAAATAATCTAAAAA	492	0.5 kbp upstream
Gh1_2	TTTTTGATGGGGATAGGGTTATAG	CTCCCTACACTAATACCCTCCAAC	351	1.0 kbp upstream
Ghr	TGATTGGGAGTGTGATGTTTTAAT	CCAAACTCCTATTCCCTAAATCCT	384	1.0 kbp upstream
Ghr_2	TTTTTGTGAAGTATGGTGTTGAAA	AAAAACCATCCCTACTCCCTTAAT	393	2.0 kbp upstream
Gli1	TTTTGTTTTTAGATTTAAAGAGGGTGT	ATTTTTCTACCAACCTCATCCTCTAA	375	3.5 kbp upstream
Gnrhr	GAAAGAAGTATTGAAAGTTGTGGAGA	AAAACCAAACCATCTACTAAAATACTACAA	453	0.2 kbp upstream
Lhb	GATGAGTAGAGGGGGGATTTGTAGA	CCCTCCTACTAACTATCCACAACC	462	0.2 kbp upstream
Lhb_2	GGGATTGAATTTGGAATTTGTATT	TCTACAAATCCCCCTCTACTCATC	395	0.5 kbp upstream
Lhx4	AGGGTATTTGTTGGTGGAGTTG	AAATACATCAACACACTATTCACAAAA	486	1.5 kbp upstream
Math3	ATTAGTTGTTTATGGGATGTTTGGTAG	AACAAAACCCAAATATAATTCCTAACC	309	8.0 kbp downstream
Math3_2	GGGAAAGGGAGGAGTTAGATTG	CAAATCTCCCTAACCATCCAAA	365	1st exon
Myt1l	AATGGATTTTATGGATGGGTTG	TACCTTTTCCAACCACACAAAC	435	1st exon
Pit1	TTTGGGTAAGAAGGAAGATAGTTTAGA	CTAACCATCTCTAAACCCAACCA	432	1st exon
Pit1_2	AAAAATTGATTTAAAATTTGATAGGGAGT	TCTAAAATCTCAATCTTAAACATAATTTCC	462	2.5 kbp upstream
Prl	TTGTAGTTTTGAAATTTTTGGTTAGAAT	AAACTAATCTCCAAATCTCACTTTCATT	450	0.2 kbp upstream
Prl_2	TATGGAAGAGGTTTGGGAATTAGATA	TCTAACCAAAAATTTCAAAACTACAAAA	397	0.5 kbp downstream
Rara	GTTTTGGTAGGGGTGGGATT	TCTTCCCCTCACTTTCCCTAA	349	0.5 kbp downstream
Rxr	TGGTGAGAGAGGGTAAGTGGA	TTCACACCCAAAACCCAACT	386	1.5 kbp downstream
Shh	GGTTAGGTAGGGGTTAAGGGAGA	ACACACACCACCCCCATAAT	439	1.5 kbp upstream
Tbx19	TTAGGTGGGAGAGAGTTTGTTTTT	TCCTCCTAATCTCTAACCCTTAAACA	427	0.5 kbp upstream
Trhr	AAGATGGTGATTATTTGGTATTTTAGG	TTTCCATTAAACACTCTCAAAACAA	447	0.5 kbp upstream
Wnt3a	AGAGGGAGAAAGGGAAGGTATTAG	CCAAAAACTAAACTATACCAAATAAACA	463	0.5 kbp upstream
Wnt3a_2	GGAGAGGTAAAGAGGGAGAGAAAG	CTCCCATCTAAATCTCCTCCAAC	394	0.5 kbp downstream
Sall4	ATTGAGTTTGAAGAATGGTGAATGT	CCAATTACTCCAACAATACCCACTA	406	0.5 kbp upstream
Meg3	TTTTTATAGGGAGATGGTTTGTGTT	ACCCCAAACAAAATAACTAATACCC	304	0.5 kbp upstream
Peg10	TTTGGAGAAATTAAGTTTTTAGGGTTT	CAATAAAATACCCATCAATTAACAACA	311	0.5 kbp downstream

	RT-PCR primers		
	Forward prrimer (5' -3')	Reverse prrimer (5' -3')	Size (bp)
Gh1	CAAAAGGGACCAATTCCAGA	CATTGGCAAATAGGCTGGAC	193
Pit1	TTCGGCTGACACCTTTATACCT	ACTCCATAGGTCGATGACTGGT	186
Lhb	GACTGCTGTTGTGGCTGCT	AAAGCTCAGCTCTCGGTAGGT	236
Tbx19	GGCAGAGATTCAAGGAAGTCAC	GCTTCACTTTGCTGAAGGAGAT	288
Prl	TAAAGCCAATATCTGGGGAAGA	TCCGAAGAGAGGTTATGGATGT	277
Gapdh	ACCACAGTCCATGCCATCAC	TCCACCACCCTGTTGCTGTA	452
