# Supplementary material for: Ultra-Deep Bisulfite Sequencing to Detect Specific DNA Methylation Patterns of Minor Cell Types in Heterogeneous Cell Populations: An Example of the Pituitary Tissue
Source: PLoS One. 2016 Jan 11;11(1):e0146498. doi: 10.1371/journal.pone.0146498 (PMC4709138; doi:10.1371/journal.pone.0146498)
Supplement: S2 Table — *The 37 pituitary-related genes with 100 or more reads obtained in each of the triplicate MiSeq sequencing runs were selected for DNA methylation analysis. For PFF, 36 genes out of the 37 pituitary-related genes gave reproducible sequencing data, and the 36 genes were used for DNA methylation analysis. **Mean ± SE. (PDF) [file pone.0146498.s006.pdf]

S2 Table. Summary of Miseq sequencing data.

	Number of analyzed genes*	Number of reads**	Number of genes sequenced more than 1,000 reads
Pituitary #1	37	5,897 ± 1,505	30
Pituitary #2	37	6,321 ± 1,484	28
Liver	37	3,832 ± 709	28
Brain	37	4,076 ± 914	25
PFF	36	4,988 ± 1,271	30

* The 37 pituitary-related genes with 100 or more reads obtained in each of the triplicate MiSeq sequencing runs were selected for DNA methylation analysis. For PFF, 36 genes out of the 37 pituitary-related genes gave reproducible sequencing data, and the 36 genes were used for DNA methylation analysis.

** Mean ± SE.
