# Supplementary material for: Ultra-Deep Bisulfite Sequencing to Detect Specific DNA Methylation Patterns of Minor Cell Types in Heterogeneous Cell Populations: An Example of the Pituitary Tissue
Source: PLoS One. 2016 Jan 11;11(1):e0146498. doi: 10.1371/journal.pone.0146498 (PMC4709138; doi:10.1371/journal.pone.0146498)
Supplement: S3 Table — The numbers of reads and Hypo-allele ratios of two pituitary (#1 and #2) and one liver sample from two independent experiments (Exps. 1 and 2) are described for the five genes. (PDF) [file pone.0146498.s007.pdf]

S3 Table. Summary of Hypo-allele analysis of the five T-DMRs of pituitary cell type-restricted genes shown in Fig. 4.

		Pituitary #1		Pituitary #2		Liver	
		Number of reads	Hypo-allele (%)	Number of reads	Hypo-allele (%)	Number of reads	Hypo-allele (%)
Gh1	Exp. 1	2,629	17.5	3,294	17.3	1,597	5.3
	Exp. 2	3,304	12.8	12,505	12.1	3,901	7.4
Prl	Exp. 1	1,107	22.7	721	34.8	172	7.0
	Exp. 2	5,338	25.8	1,564	47.1	4,892	4.4
Lhb	Exp. 1	1,335	15.7	2,437	41.2	1,133	0
	Exp. 2	1,692	9.3	8,003	29.5	2,672	0
Tbx19	Exp. 1	1,024	11.6	1,361	13.3	1,131	0
	Exp. 2	820	23.2	3,068	13.2	1,493	0
Pit1	Exp. 1	1,978	63.1	1,875	51.5	1,460	6.0
	Exp. 2	1,968	59.6	3,984	82.2	2,321	0

The numbers of reads and Hypo-allele ratios of two pituitary (#1 and #2) and one liver sample from two independent experiments (Exps. 1 and 2) are described for the five genes.
